# Olfactory Jump Reflex Habituation in *Drosophila* and Effects of Classical Conditioning Mutations

**DOI:** 10.1080/01677060701247508

**Published:** 2007-10-15

**Authors:** Zoltan Asztalos, Neeraj Arora, Tim Tully

**Affiliations:** Cold Spring Harbor Laboratory, Cold Spring Harbor, NY

**Keywords:** *Drosophila*, *Dunce*, Habituation, Learning, Memory, Olfactory jump reflex, *Rutabaga* mutants

## Abstract

Habituation is a nonassociative learning mechanism, in which an initial response toward repeated stimuli gradually wanes. This is amongst the simplest and most widespread forms of behavioral plasticity. So far, neither the underlying molecular mechanisms nor the precise neural networks of habituation are well understood. We have developed a novel paradigm to quantify habituation of the olfactory jump reflex in *Drosophila*. We present data demonstrating several behavioral properties of this phenomenon, generally observed in other species. We also show that the *dunce* and *rutabaga* memory mutants behave abnormally in this assay, suggesting that this assay might be used in behavioral screens for new mutants with defects in this simpler form of behavioral plasticity.

## INTRODUCTION

Habituation is a nonassociative learning mechanism whereby an initial response wanes towards repeated stimuli. We can consider habituation as a natural adaptation process to environmental stimuli. This is amongst the simplest and most widespread forms of behavioral plasticity. It has been observed from protozoan through mollusks (Wyers et al., 1973) to mammals, and including humans (Deutsch & Deutsch, 1973).

Decades ago, a set of criteria has been compiled to define differences between sensory adaptation, effector fatigue and genuine habituation (Groves & Thompson, 1970; Thompson & Spencer, 1966), but the underlying molecular mechanisms of habituation still are not well understood. The best-studied system to date is the siphon withdrawal reflex in a marine mollusk, *Aplysia californica*. Study of this circuit has shown that monosynaptic depression at presynaptic terminals of mechanoreceptor cells at least are partially responsible for diminishing responses during repeated tactile stimulation (Castellucci et al., 1970; Pinsker et al., 1970). This finding is consistent with data from work on crayfish (Krasne, 1969; Zucker, 1972), *C. elegans* (Wicks & Rankin, 1997), *Aplysia* (Rayport & Schacher, 1986) and rat cultured neurons (McFadden & Koshland, 1990). This response depression involves a reduced Ca^++^ influx (Edmonds et al., 1990; Klein et al., 1980) and decreased presence of synaptic vesicles at active zones of sensory neuron presynaptic terminals, leading to decreased neurotransmitter release (Bailey & Chen, 1988). Additional studies suggest that monosynaptic depression cannot be the only mechanism to achieve behavior habituation, and other parallel neural mechanisms, such as long-term depression, might be present, as well (Peretz et al., 1976; Stopfer & Carew, 1996).

Isolating new learning mutants is a potentially effective way to uncover the molecular pathways underlying learning and memory formation. The handful of genetic screens performed so far (Aceves-Pina et al., 1983; Boynton & Tully, 1992; Dubnau et al., 2003; Dura et al., 1993) employed associative learning assays that are labor intensive and yielded a limited number of mutants. As a possible alternative, we have developed a versatile jump reflex habituation paradigm, suitable for analyzing behavior properties of habituation in *Drosophila*, which can be used as an effective screen for mutants affecting simpler forms of behavioral plasticity.

It is already established that learning mutants isolated in instrumental or classical conditioning paradigms, as *dunce* (Dudai et al., 1976) amnesiac (Quinn et al., 1979), *rutabaga* (Aceves-Pina et al., 1983), and *Su-var*(3)601 (Asztalos et al., 1993) are affected in nonassociative learning as well (Asztalos et al., 1993; Corfas & Dudai, 1989; Duerr & Quinn, 1982; Engel & Wu, 1996; Rees & Spatz, 1989; Vaias et al., 1993; Wittekind & Spatz, 1988). In line with those findings in our new jump reflex habituation paradigm *rutabaga* has reduced rate of habituation—conforming the potentials of our essay in detecting novel habituation mutants.

## MATERIALS AND METHODS

### Stocks and Maintenance

The fruit fly *Drosophila melanogaster* was used in all experiments. The Canton-Special (Canton-S) strain was used as wild-type, and hemizygous males from the following mutant lines were tested: *rutabaga^2080^* (*rut^2080^*; Levine et al., 1995), *dunce^1^* (*dnc^1^*; Duerr & Quinn, 1982) and *dunce^M14^* (*dnc^M14^*; Mohler & Carroll, 1984).

All strains were raised in plastic bottles on standard cornmeal medium in which four different mixtures of antibiotics were rotated every generation. The stocks were maintained at 25°C, 50% relative humidity on a 16=8 h light=dark cycle. When breading flies for behavioral experiments, 10 pairs of flies were set up in small glass vials on cornmeal medium always containing the same mixture of antibiotics, transferred to new vials every two days and maintained as above. Male flies eclosing from glass vials were collected under light CO_2_ anesthesia on the same day and stored in vials as above in groups of about 20. Test flies were 2–3 days old.

The genetic background of the mutant stocks was replaced with that of the control, Canton-S, by outcrossing them at least seven times to “cantonized” FM7a flies.

### Chemicals

Highest purity benzaldehyde (FLUKA, Buchs SG, Switzerland) and heavy mineral oil (Fisher Scientific, Pittsburgh, PA, USA) were used.

### Jump Reflex Habituation Assay

The olfactory jump reflex habituation machine was designed and manufactured by General Valve Company, now part of Parker Hannifin Corporation, Hollis, NH USA. The jump reflex habituation software was made by Omnitech Inc. Sioux Falls, SD, USA and is currently sold by AccuScan Instruments, Inc. Columbus, Ohio, USA.

**Figure 1:**
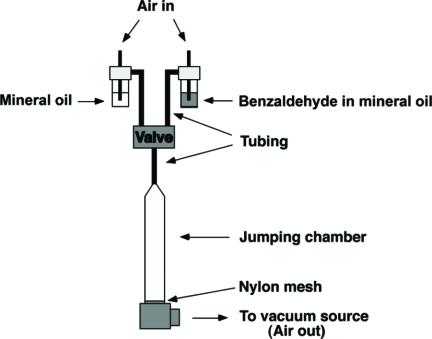
Apparatus for habituation of the olfactory jump response. For details, see Materials and Methods.

For habituation training, single males were housed in transparent plastic test tubes (Figure 1). The test tubes were inserted into an aluminum base with small holes in it. A vacuum source was connected to the base creating a continuous airflow (1000 ml/min). Odorant was delivered by a computer controlled 3-way solenoid valve which switched the air current from air, which was traveling through mineral oil (control), to air which bubbled through a certain concentration of benzaldehyde (BA) in mineral oil. Each training trial consisted of a four-second odor presentation with varying inter-trial interval (ITI). Response to odorant was monitored as to whether or not the fly jumped during the four-second odor presentation or in the following three seconds and deemed habituated when it failed to jump in four consecutive trials (no-jump criterion). A habituation score was expressed as the number of trials required to reach the no-jump criterion (trials to criterion; TTC).

Failure to respond to the odor pulse caused by fatigue was monitored by dishabituating flies that reached no-jump criterion. Upon reaching criterion, the chamber containing the fly was vortexed for 75 s, returned to the habituation apparatus, the odor then was presented for four seconds and the fly was scored for a jump response. Thus the dishabituation test occurred two minutes after the last training trial. Dishabituation score (DIS) is the percentage of flies that jumped to the odor test-trial after vortexing. To test for spontaneous recovery (a measure of memory loss), a separate set of flies were left undisturbed during the two-minute period, presented with odor and then subjected to an odor test-trial. Spontaneous recovery score (SR) is the percentage of these flies that jumped to the odor test-trial.

Experiments were run in an environmentally controlled room at 25C and at 70–80% relative humidity.

### Statistical Analyses

JMP 3.1 statistical software (SAS Institute Inc., Cary, NC USA) was used for data analysis.

As TTCs did not show normal distribution (data not shown), we employed a non-parametric Wilcoxon/Kruskal-Wallis test to compare mutant data with those of wild-type (α = 0.05).

To obtain Spontaneous Recovery (SR) and Dishabituation (DIS) scores, means of single-day data were calculated. In accordance with the central limit theorem, the distribution of these means proved normal (data not shown). Mean and Standard Error of the Mean (SEM) of the daily means were calculated. Student's t-test was used to estimate the difference between mutant and control data (α = 0.05).

To compare jump responses in 1- and 10-min ITI habituation experiments and in olfactory acuity experiments, ANOVA was applied.

## RESULTS

We studied the different variables of the olfactory jump response and its decrement to repeated odor presentations for optimizing the habituation paradigm, and to demonstrate that the decrease in jump response reflects habituation rather than sensory or motor fatigue.

### Olfactory Jump Response

**Figure 2:**
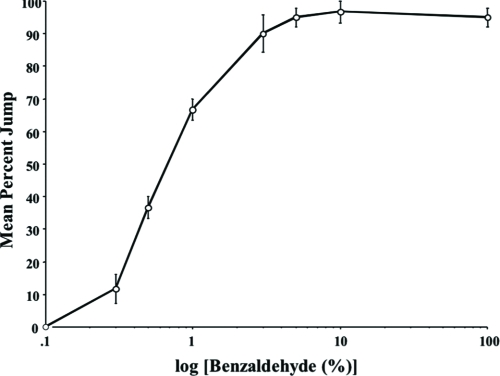
Olfactory acuity of jump response. Eight concentrations of benzaldehyde (BA) were prepared in mineral oil by serial dilution—0.1%, 0.3%, 0.5%, 1%, 3%, 5%, and 100% (undiluted BA). Each fly was scored for its jump response (1 = yes, 0 = no) to a single odor presentation in a fresh chamber. Twenty wild-type (Canton-S) males were tested per concentration per day, and the mean percent jump response was calculated for each BA concentration for the day. The results shown are the means of daily means ± Standard Standard Error of the Mean (SEM). n = 3 daily means per group.

To a range of benzaldehyde (BA) concentrations fruit flies show a jump response (Figure 2). At a concentration of 3% BA, this response reaches its maximum and does not change with higher concentrations. We have chosen 5% BA as the “standard” concentration for habituation experiments, as it is low but still sufficient to evoke a maximum percentage of initial jump response reliably.

### Decrement of Jump Response to Repeated Odor Presentations

**Figure 3:**
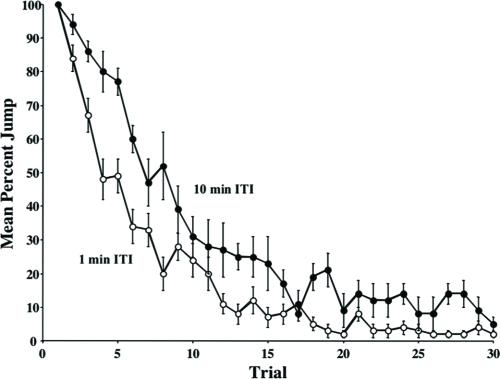
Decrement of jump response to repeated odor presentations. Individual flies were given 30 odor presentations (5% BA) at either a 1-min or a 10-min intertrial interval (ITI). 16 flies were tested per ITI per day and a mean jump responses were calculated for both it is for that day. The results shown are the means of daily means ±SEM. N = 4 daily means per group. With a 10-min ITI, the jump response wanes significantly more slowly than that with a 1-min ITI (two-way ANOVA, *p* < 0.001).

Upon repeated presentation of 5% BA, the initial high levels of jump response gradually wanes—as we will discuss later, because the fly habituates. The rate of this response decrement depends on the intertrial interval (ITI; Figure 3). At a 10-min ITI, the jump response declines significantly slower than that at a 1-min ITI (two-way ANOVA, ITI effect *p* < 0.001).

A fly can be considered “habituated” when it reaches the no-jump criterion. After this point, the probability of a subsequent jump response is quite low, but not zero (data not shown). Consequently, each fly can be assigned a habituation score based on the number of trials to criterion (TTC).

### Spontaneous Recovery

**Figure 4:**
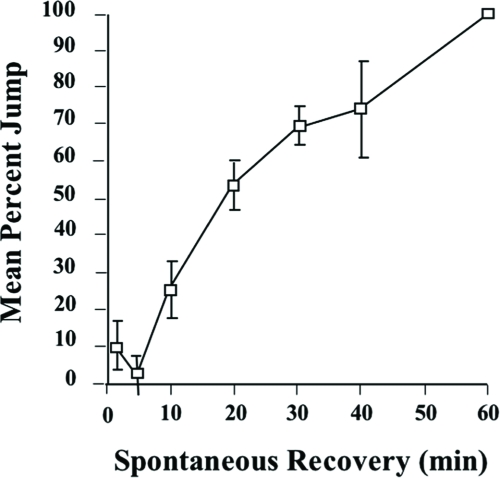
Spontaneous recovery of the jump response. Flies were trained with a 1-min ITI and then subjected to an odor test-trial at 2, 5, 10, 20, 30, 40, and 60 min after reaching a no-jump criterion (see Materials and Methods). 12 flies were tested per recovery interval per day. The results shown are the means of the daily means ±SEM. n = 5 daily means per group.

Habituation is a readily reversible process (Groves & Thompson, 1970; Thompson & Spencer, 1966). If we monitor jump response at different times after flies reach the no-jump criterion, the jump response slowly recovers and reaches its original maximum at around 60 min after the last training trial (Figure 4). Such spontaneous recovery (SR) is a defining characteristic of habituation, distinguishing it from possible damaging effects of training.

### Effect of Inter-Trial Interval on Habituation Scores

**Figure 5:**
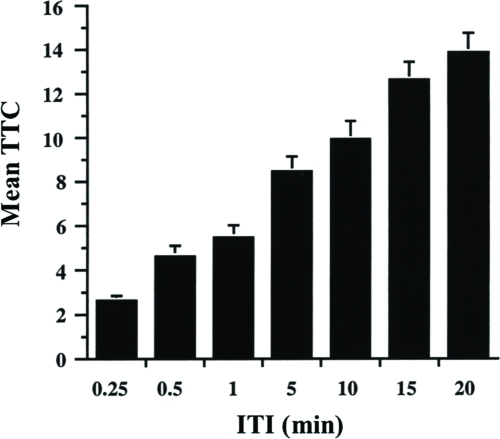
Effect of intertrial interval on jump response decrement. Flies were trained with one of seven different ITIs—0.25, 0.5, 1, 5, 10, 15, and 20 min. 8 to 16 flies were tested per ITI per day (experiments at longest ITI frequently lasted very long times), and each group was repeated over four days. The results shown are the mean TTC score ±SEM per ITI. n = 34 to 64 flies per group.

To demonstrate that memory formation is part of the habituation process, we subjected flies to training protocols with different InterTrial Intervals. As shown in Figure 5, TTC increase with increasing ITI. This phenomenon can be explained either by increased memory loss between trials with longer ITIs or by less fatigue with longer ITIs.

### No Effects of Motor Fatigue

**Figure 6:**
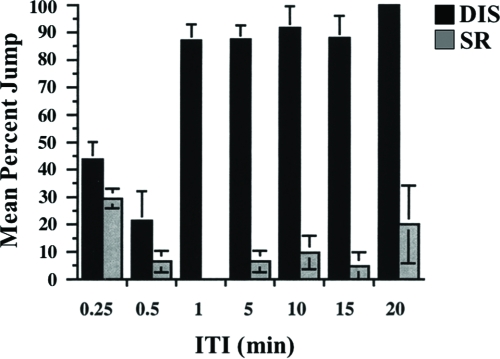
Effect of inter-trial interval on spontaneous recovery and dishabituation. Flies were trained at one of seven different ITIs—0.25,.5, 1, 5, 10, 15, and 20 min (same experiment as in Fig. 5). 4 to 8 flies were tested per SR or DIS group per ITI per day. The results shown are the means of daily means ±SEM. n = 4 daily means per group.

One of the crucial tests for habituation is the presence of dishabituation (Groves & Thompson, 1970; Thompson & Spencer, 1966). In these experiments, we applied a strong mechanical stimulus (75 s of vortexing) immediately after the fly achieved its no-jump criterion and then subjected it to an odor test-trial afterwards. For ITIs of 1-min and longer, dishabituation scores are significantly higher than that of spontaneous recovery scores (Figure 6). If sensory or motor fatigue produced the observed jump response decrement during training, then we would not expect subsequent jump responses to the test-trial to be any higher for the dishabituation group (DIS) than for the spontaneous recovery group (SR). Alternatively, if the test-trail jump response is appreciably higher for DIS than for SR, then we can argue against the presence of fatigue and for the occurrence of dishabituation. For ITIs shorter than 1 min, in fact, DIS scores are lower than for longer ITIs and similar to SR scores, suggesting the presence of fatigue at these shorter ITIs. For this reason, we uses ITIs of 1 min or longer for further habituation experiments.

### Effect of Odor Intensity on Habituation Scores

**Figure 7:**
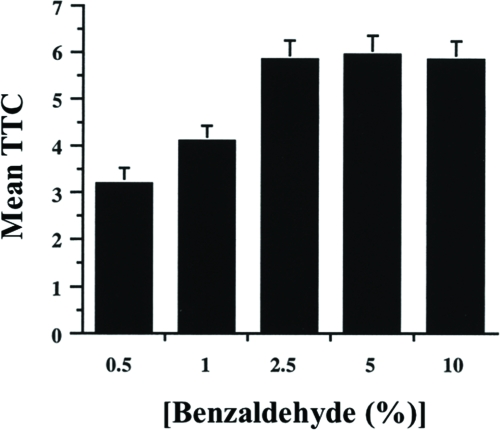
Effect of odor concentration on jump response decrement. Flies were trained with a 1-min ITI at one of five different benzaldehyde (BA) concentrations—0.5%, 1%, 2.5%, 5%, and 10%. 30 flies were tested per BA concentration per day, and repeated over four days. The results shown are the mean TTC score ±SEM for each BA concentration. n = 120 flies per group.

To show that the jump response decrement is due to habituation rather than sensory adaptation, we measured habituation at different benzaldehyde concentrations. In case of olfactory adaptation, one expects that with increasing BA concentrations the probability of response declines faster, yielding lower TTC scores for higher odor intensities. The opposite is expected for habituation, where response decrements to higher stimulus intensities take longer (Groves & Thompson, 1970; Thompson & Spencer, 1966). We observed the latter in our experiments (Figure 7). TTC scores increased with higher BA concentrations and reached a maximum at about 2.5% BA. This observation supports the notion that the jump response decrement observed during training also does not result from appreciable sensory adaptation to olfactory cues.

### *Rutabaga* and *Dunce* Mutant Show Defects In Olfactory Jump Response

To explore the potential usefulness of this habituation paradigm to identify and characterize learning mutants, we characterized three strains of two well-known memory mutants, *rutabaga* (*rut*) and *dunce* (*dnc*). Both of these mutants have been reported to show abnormal responses in other assays of habituation (Corfas & Dudai, 1989; Duerr & Quinn, 1982; Engel & Wu, 1996; Gailey et al., 1991; Rees & Spatz, 1989).

**Figure 8:**
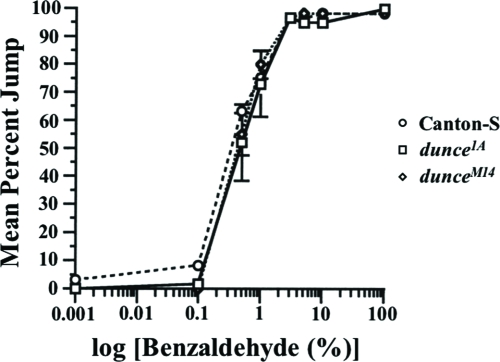
Olfactory acuity in wild-type (Canton-S) and mutant (*dunce*) flies. Eight concen trations of BA were prepared in mineral oil by serial dilution—0.001%, 0.1%, 0.5%, 1%, 3%, 5%, 10%, and 100% (undiluted BA). Canton-S, *dnc^1A^* and *dnc^M14^* flies were tested on the same days. Each fly was score for its jump response to a single odor presentation in a fresh chamber. 20 of each line were tested per concentration per day, and the mean jump response was calculated for each BA concentration each day. The results shown are the means of daily means ±SEM. n = 3 daily means per group.

We studied the *dunce^1A^* (*dnc^1A^*; Duerr & Quinn, 1982), *dunce^M14^* (*dnc^M14^*; Mohler and Carroll, 1984) and *rutabaga^2080^* (*rut^2080^*; Levin et al., 1992) alleles. Homozygotes of both *dunce* alleles show normal levels of initial jump response to different benzaldehyde concentrations (Figure 8, two-way ANOVA, genotype effect *p* = 0.1094), as does *rut^2080^* (data not shown, two-way ANOVA, genotype effect *p* = 0.1186).

**Figure 9:**
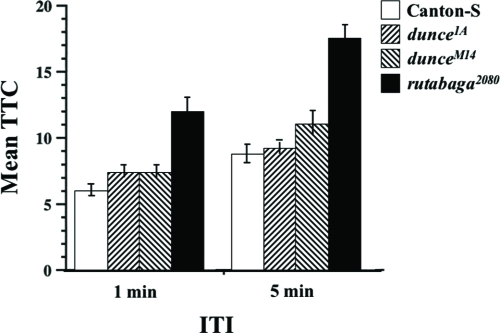
*Dunce* and *rutabaga* mutants habituate more slowly than normal. Canton-S, *dnc^1A^*, *dnc^M14^*, and *rut^2080^* flies were trained with either a 1-min or a 5-min ITI using 5% BA. The lines were tested together each of several days. The results shown are mean TTC score ±SEM per ITI per line. n = 120 flies and 80 flies for the 1-min and 5-min ITIs, respectively.

**Figure 10:**
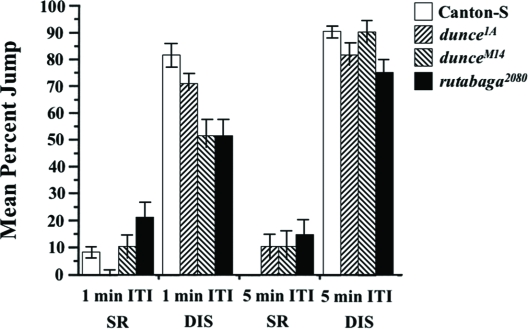
Spontaneous recovery and dishabituation in *dunce* and *rutabaga* mutants are defective. Flies were trained with either a 1-min or a 5-min ITI using 5%BA (same experiment as in Fig. 9). After flies reached their no-jump criterion, spontaneous recovery (SR) or dishabituation (DIS) were quantified with a subsequent odor test trial (see Materials & Methods). For the 1-min ITI groups, 8 to 12 flies were tested for SR or DIS per genotype per day. For the 5-min ITI groups, 2 to 6 flies were tested for SR or DIS per genotype per day. The results shown are the means of daily means ±SEM. N = 5 and 7 daily means per group for the 1-min and 5-min ITIs, respectively.

With a 1-min ITI, homozygotes of all three mutant alleles show significantly greater TTC scores (habituate more slowly) than do wild-type flies (Figure 9, Wilcoxon non-parametric test, *p* = 0.0387 for *dnc^1A^*, *p* = 0.0273 for *dnc*^*M14*^ and *p* < 0.0001 for *rut^2080^*). Dishabituation scores after training with a 1-min ITI for *dnc^M14^* and *rut^2080^* mutants, however, are significantly lower than that of Canton-S (Figure 10, t-test, *p* = 0.0417 and *p* = 0.0296, respectively), suggesting a slower recovery from fatigue in these mutants.

To examine this further, we trained mutant and normal flies with a 5-min ITI, expecting that mutants would have more time to recovery from any fatigue effects. Here again, *dnc^M14^* and *rut^2080^* mutants yielded TTC scores significantly higher than Canton-S flies (Figure 9, Wilcoxon non-parametric test, *p* < 0.0001 and *p* < 0.01). Surprisingly, *dunce^1A^* mutants displayed TTC scores no different from normal when trained with a 5-min ITI (Figure 9, Wilcoxon non-parametric test, *p* = 0.5712). As expected, DIS scores for all mutants are higher after training with a 5-min ITI compared to training with a 1-min ITI, and only that for *rut^2080^* mutants is significantly lower than the corresponding DIS score for Canton-S flies (Figure 10, t-test, *p* = 0.0354). Together, these observations support the interpretation that fatigue effects in mutants likely reduced their TTC and DIS scores. It remains difficult to explain why *dunce^1A^* mutants appear abnormal after training with a 1-min ITI but not a 5-min ITI.

## DISCUSSION

Previous reports have indicated that fruit flies display a jump response to odors (McKenna et al., 1989) and that this olfactory jump response wanes with repeated odor presentation (Tully & Koss, 1993). Here, we demonstrate that habituation contributes to this response decrement with behavioral properties similar to other species (Groves & Thompson, 1970; Thompson & Spencer, 1966). Higher stimulus intensities yield slower response decrement (Figure 7). An initially high level of jump response (Figure 2) wanes with repeated odor presentations (Figure 3). The decremented response then recovers spontaneously over time if a fly is left undisturbed (Figure 4). If decremented flies are exposed to a strong, noxious stimulus (vortexing), dishabituation occurs, and their responses to an odor test-trial are higher than those after spontaneous recovery (see Figure 4). The response decrement is slower with longer ITIs (Figure 5). These observations constitute a classic characterization of habituation, a simple form of nonassociative behavior plasticity.

During a habituation procedure, sensory and motor fatigue also might contribute to the observed response decrement. Traditionally, two behavioral properties have been used to distinguish these phenomena. First is the demonstration of dishabituation. A decremented response recovers immediately after the animal is exposed to a strong stimulus, especially in another sensory modality, but such rapid recovery cannot occur in the presence of fatigue. Evidence for the latter is apparent in our study, when wild-type flies are trained with ITIs shorter than 1 min (Figure 6) or when mutant flies are trained even with a 1-min ITI (Figure 10). Second is the relation between stimulus intensity and response decrement. Increasing (sensory) fatigue, produced by increasing stimulus intensity, would cause a faster response decrement, while the opposite would be expected in the case of habituation. Our experiments confirmed the latter using a 1-min ITI (Figure 7).

The memory mutants *dunce* (Dudai et al., 1976), which are impaired in cAMP phosphodiesterase, and *rutabaga* (Aceves-Pina et al., 1983), which are defective in Ca^2+^/calmodulin-dependent adenylate cyclase (Levin et al., 1992), were isolated in an operant conditioning (associative learning) paradigm (Quinn et al., 1974) and also have been shown to be defective in a classical conditioning (associative learning) paradigm (Tully & Quinn, 1985). These mutants also have been show to be defective in nonassociative learning. Employing a proboscis-extension reflex paradigm (Duerr & Quinn, 1982) claimed that *dunce* and *rutabaga* habituate more slowly than wild-type flies. In a cleaning reflex preparation, Corfas and Dudai (Corfas & Dudai, 1989) showed that habituation of *rutabaga* and *dunce* were normal but their memories decayed more quickly than that of wild-type flies. In an electrophysiological assay, Engel and Wu (Engel & Wu, 1996) found a reduced rate of habituation in *rutabaga* mutants, whereas *dunce* mutants showed a moderate increase in habituation rates. Finally, habituation of the landing response was found to be faster than normal in *dunce* and *rutabaga* mutants (Rees & Spatz, 1989; Wittekind & Spatz, 1988). These somewhat varied observations likely result from differing contributions of *bona fide* habituation and fatigue among these assays.

In our habituation paradigm, *rutabaga^2080^* (P-element mutant; Levin et al., 1992), *dunce^1^* (*dnc^1^*, hypomorphic EMS mutant; Duerr & Quinn, 1982) and *dunce^M14^* (*dnc^M14^*, amorphic EMS mutant; Mohler & Carroll, 1984) were tested. The mutations were outcrossed to our wild-type genetic background, so their mutant phenotypes likely results from the documented genetic lesions or possibly from very closely linked second-site effects. Olfactory acuity of these mutants was normal (Figure 8, and data not shown), indicating that they can sense and respond normally to benzaldehyde. With the exception of *dnc^1A^* trained with a 5-min ITI, habituation generally was slower than normal for the memory mutants. This observation supports a general interpretation that habituation is slower with increasing ITI because of greater memory loss between each trial. Thus, memory decay is greater than normal for these memory mutants at a given ITI (see SR in Figure 10), thereby yielding higher TTC scores which is particulary evident for *rut* mutants (Figure 9).

The abnormally low DIS scores for mutants, which nonetheless diminishes with increasing ITI (Figure 10), suggest a greater fatigue component to the mutants' jump responses. Such fatigue would serve to decrease TTC scores; thus, mutant defects in the habituation process might even be greater than observed. Although the existence of these kinds of mutants could be predicted on the basis of results obtained in *C. elegans* (Broster & Rankin, 1994; Rankin & Broster, 1992), further investigations are needed to clarify these potential component effects on jump response. Nonetheless, our data indicate that the cAMP pathway plays a significant role in a habituation of the olfactory jump response. More generally, this habituation assay may be suitable for a behavioral screen for mutants with defects in this simpler form of behavioral plasticity.
